# Novel *Rickettsia* genotypes in ticks in French Guiana, South America

**DOI:** 10.1038/s41598-020-59488-0

**Published:** 2020-02-13

**Authors:** Florian Binetruy, Marie Buysse, Roxanne Barosi, Olivier Duron

**Affiliations:** Laboratoire Maladies Infectieuses et Vecteurs: Ecologie, Génétique, Evolution et Contrôle (MIVEGEC), Centre National de la Recherche Scientifique (CNRS) - Institut pour la Recherche et le Développement (IRD) - Université de Montpellier (UM), 911 Avenue Agropolis, F-34394 Montpellier, France

**Keywords:** Infectious-disease epidemiology, Bacterial infection

## Abstract

*Rickettsia* are obligate intracellular bacteria often associated with ticks and best known for causing human diseases (rickettsiosis), including typhus fever and sporadic cases of serious infection. In this study, we conducted a large survey of ticks in French Guiana to understand the overall diversity of *Rickettsia* in this remote area largely covered by dense rainforests. Out of 819 individuals (22 tick species in six genera), 252 (30.8%) samples were positive for *Rickettsia* infection. Multilocus typing and phylogenetic analysis identified 19 *Rickettsia* genotypes, but none was 100% identical to already known *Rickettsia* species or strains. Among these 19 genotypes, we identified two validated *Rickettsia* species, *Rickettsia amblyommatis* (spotted fever group) and *Rickettsia bellii* (bellii group), and characterized a novel and divergent *Rickettsia* phylogenetic group, the guiana group. While some tick hosts of these *Rickettsia* genotypes are among the most common ticks to bite humans in French Guiana, their potential pathogenicity remains entirely unknown. However, we found a strong association between *Rickettsia* genotypes and their host tick species, suggesting that most of these *Rickettsia* genotypes may be nonpathogenic forms maintained through transovarial transmission.

## Introduction

Members of the *Rickettsia* genus are obligate intracellular bacteria of eukaryotes^1–3^. The best known *Rickettsia* species are major human pathogens that include the etiological agents of the epidemic typhus, *R. prowazekii*, the Rocky Mountain spotted fever, *R. rickettsii*, and the flea-borne spotted fever, *R. felis*^1,4,5^. Most of these pathogenic *Rickettsia* species have a zoonotic life cycle and are transmitted by blood-feeding arthropods such as ticks, mites, lice and fleas, which commonly serve as ecological bridges for transmission from wildlife to humans and domestic animals^1,4,5^. However, members of the *Rickettsia* genus are actually more widespread than previously recognized: ecological surveys are uncovering substantial *Rickettsia* diversity associated with blood-feeding arthropods but also with non-blood-feeding arthropods, protozoa, algae and plants^1–3^.

There are currently more than 30 recognized *Rickettsia* species but the advent of multilocus sequence typing (MLST) and molecular phylogenetics has recently led to the description of several new putative species^1,2,4,6,7^. Historically, *Rickettsia* were classified into a few major groups based on serological characteristics, but subsequent DNA sequencing led to classification of at least 10 distinct phylogenetic groups^1,2,6^. Three *Rickettsia* groups – the spotted fever group, the typhus group and the transitional group – are the subject of intensive study since they all include major pathogenic species and are commonly found in blood-feeding arthropods^1^. In addition, another *Rickettsia* group widely found in ticks but also in many other arthropods, the bellii group, is also commonly studied. This group is thought to be basal to the three other major *Rickettsia* groups and is largely composed of nonpathogenic *Rickettsia* species and strains^1–3,7^.

Despite considerable research effort, the diversity of *Rickettsia* remains largely unknown in most remote geographic regions and in undersampled arthropod taxa. Interestingly, a recent survey reported the presence of 22 tick species in French Guiana^8^, a vast equatorial land located on the northeast coast of South America and mostly covered by dense rainforests including old-growth forests, which are biodiversity hotspots. French Guiana’s human population (ca. 250,000 inhabitants) is concentrated principally in a handful of towns spread along the coastline and main rivers, while the interior is largely uninhabited. We know little on the presence, diversity and effect of *Rickettsia* on ticks in this region. Only one *Rickettsia* species of the spotted fever group, *Candidatus* Rickettsia wissemanii, has been documented in bat soft ticks *Ornithodoros hasei* caught in French Guiana^9^. Some of the tick species of French Guiana are also present in adjacent countries such as Brazil, but the presence of *Rickettsia* has been investigated there in a few of these species including the Cayenne tick *Amblyomma cajennense* and the arboreal tick *A. longirostre*^7,10–14^.

In this study, we conducted a wide molecular survey of *Rickettsia* in ticks in French Guiana. This survey included 819 field specimens belonging to 22 tick species of the 33 known from French Guiana^8^. We further used MLST, including *gltA*, *coxA*, *atpA*, *ompB* and 16S rRNA gene sequences, and phylogenetics for the description of these *Rickettsia* infections. Lastly, we examined and discussed their genetic proximity with known *Rickettsia* species and strains.

## Results

### Detection of *Rickettsia*

**Figure 1:**
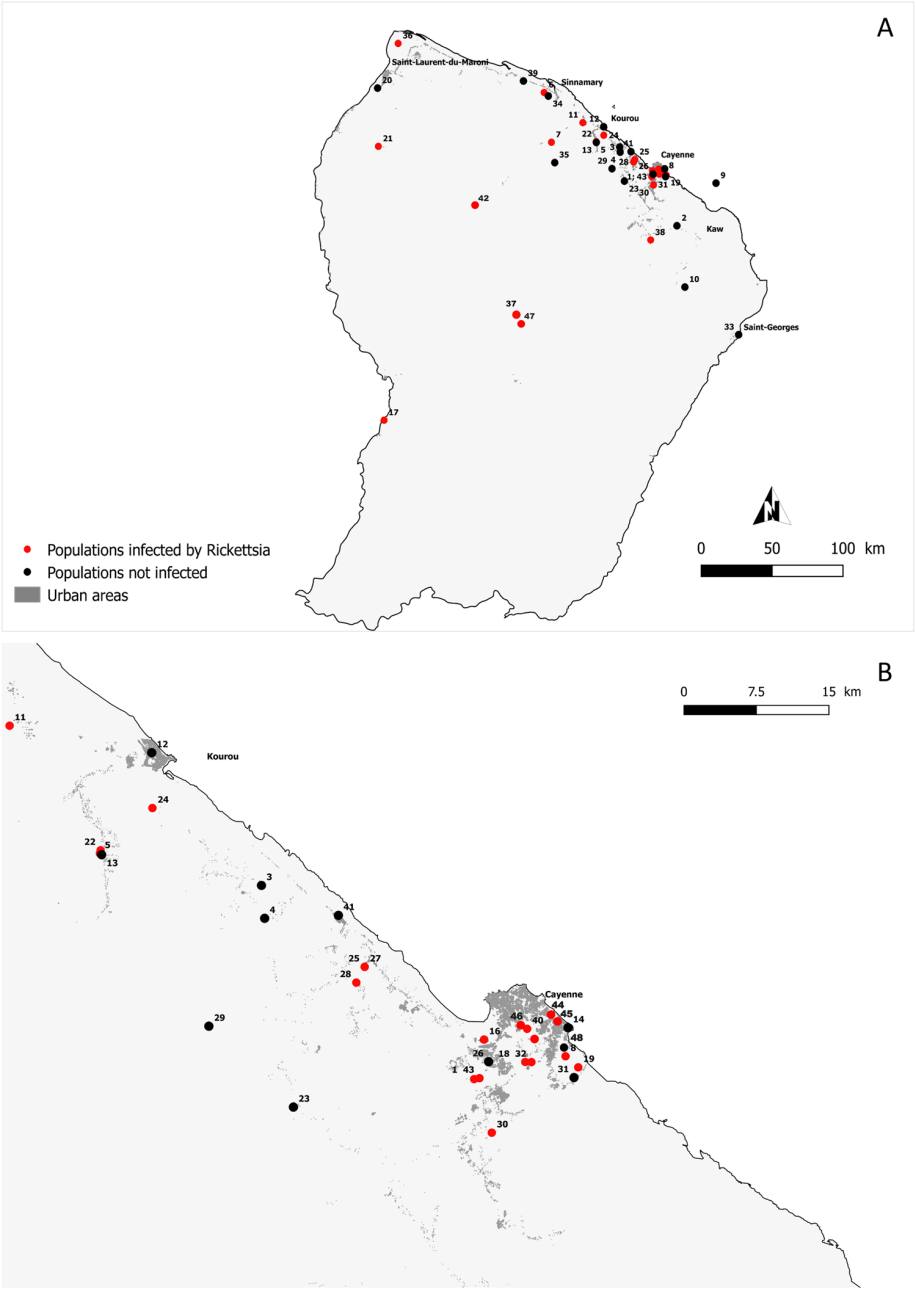
Location of sampling sites in French Guiana. Localities are represented by dots and numbers correspond to the sampling locality number given in Table S1. (**B**) is the magnification of the area bounded by the translucid grey rectangle in (**A**). Red and black dots indicate sampling localities where *Rickettsia* was detected or not, respectively.

Using total tick DNA extracts, we applied a high-throughput 16S rDNA sequencing approach to characterize the whole bacterial diversity in each tick specimen and then to detect the presence of *Rickettsia*. We assayed for the presence of *Rickettsia* in 819 individual ticks collected in French Guiana and belonging to six genera and 22 species: *Amblyomma* (16 species, 686 specimens), *Rhipicephalus* (2 species, 16 specimens), *Ixodes* (1 species, 6 specimens), *Dermacentor* (1 species, 97 specimens), *Haemaphysalis* (1 species, 8 specimens) and *Ornithodoros* (1 species, 6 specimens) (Fig. 1 and Table 1).

**Table 1:** List of tick species and sampling localities included in the analysis, with details on the sample size (*n*), and the prevalence of *Rickettsia*.

Ticks Species		Locality (# on Fig. 1)	Questing/Feeding ticks	*n* examined	*n Rickettsia*-positive - %	
**Ixodidae (hard ticks):**						
1-	*Amblyomma cajennense* sensu stricto (Fabricius, 1787)	7 localities (#1,2,6,7,11,13,43)	Questing	351	88	25%
2-	*A. calcaratum* Neumann, 1899	1 locality (#22)	Feeding	1	0	0%
3-	*A. coelebs* Neumann, 1899	4 localities (#2,6,7,34)	Questing	14	4	29%
4-	*A. dissimile* Koch, 1884	5 localities (#1,17,36,44,45)	Feeding	24	16	67%
5-	*A. geayi* Neumann, 1899	5 localities (#8,19,21,32,38)	Feeding	16	10	62%
6-	*A. goeldii* Neumann, 1899	1 locality (#38)	Feeding	5	4	80%
7-	*A. humerale* Koch, 1844	4 localities (#13,15,42,47)	Feeding	10	5	50%
8-	*A. latepunctatum* Tonelli-Rondelli, 1939	2 localities (#7,23,33)	Questing	4	4	100%
9-	*A. longirostre* (Koch, 1844)	14 localities (#8,19–31)	Feeding	130	106	82%
10-	*A. naponense* (Packard, 1869)	3 localities (#2,33,42)	Questing and Feeding	5	1	20%
11-	*A. oblongoguttatum* Koch, 1844	3 localities (#6,7,11)	Questing and Feeding	95	0	0%
12-	*A. pacae* Aragão, 1911	2 localities (#11,31)	Questing and Feeding	7	0	0%
13-	*A. romitii* Tonelli-Rondelli, 1939	1 locality (#41)	Feeding	2	0	0%
14-	*A. rotundatum* Koch, 1844	2 localities (#5,39)	Questing and Feeding	6	0	0%
15-	*A. scalpturatum* Neumann, 1906	5 localities (#5,10,11,35,37)	Questing and Feeding	8	0	0%
16-	*A. varium* Koch, 1844	5 localities (#1,16,32,43,44)	Questing and Feeding	8	5	63%
17-	*Dermacentor nitens* Neumann, 1897	1 locality (#4)	Feeding	97	0	0%
18-	*Haemaphysalis juxtakochi* Cooley, 1946	3 localities (#5,7,11)	Feeding	8	3	38%
19-	*Ixodes luciae* Senevet, 1940	3 localities (#18,40,46)	Feeding	6	6	100%
20-	*Rhipicephalus microplus* (Canestrini, 1888)	1 locality (#3)	Questing and Feeding	10	0	0%
21-	*R. sanguineus* sensu lato (Latreille, 1806)	2 localities (#12,14)	Feeding	6	0	0%
**Argasidae (soft ticks):**						
22-	*Ornithodoros capensis *sensu stricto Neumann, 1901	1 locality (#9)	Feeding	6	0	0%
			Total	819	252	31%

Of the 819 specimens, 252 (30.8%) exhibited *Rickettsia* 16S rDNA reads. The 567 remaining specimens (69.2%) were devoid of any of *Rickettsia* 16S rDNA reads but had satisfactory DNA template quality, as shown by the positive amplification of other bacterial 16S rDNA reads commonly detected in ticks (including *Coxiella*- and *Francisella*-like endosymbionts^15–18^). Of the 22 tick species examined, 10 species (6/16 *Amblyomma* species, 1/1 of *Dermacentor*, 2/2 of *Rhipicephalus* and 1/1 of *Ornithodoros*) were not infected by *Rickettsia* (Table 1 and S1). The 12 other tick species (10/16 *Amblyomma* species, 1/1 *Haemaphysalis* and 1/1 *Ixodes*) were *Rickettsia*-positive for at least one of the examined specimens (Fig. 1, Tables 1 and S1). The detection rate of *Rickettsia* did not co-vary with the screening effort, i.e., the number of examined specimens per tick species (Spearman’s rank correlation, *n* = 22, r_s_ = 0.20, *p* = 0.37): the tick species observed with *Rickettsia* infections were not those for which we examined more specimens. This is best exemplified by (1) *Rickettsia*-positive tick species for which we examined few specimens, such as *I luciae* (*n* = 6 examined specimens and all were *Rickettsia*-positive) and *A. goeldii* (*n* = 5 examined specimens and 4 were *Rickettsia*-positive), and (2) *Rickettsia*-negative tick species for which we examined a large number of specimens, such as *A. oblongoguttatum* (*n* = 95 examined specimens but none positive) and *D. nitens* (*n* = 97 examined specimens but none positive) (Fig. 1, Tables 1 and S1).

The prevalence of *Rickettsia* differed substantially between the 12 infected tick species (Fisher’s exact test, *p* = 2.10^−16^): while prevalence is low to moderate in some tick species (e.g., 4 infected specimens of the 14 examined in *A. coelebs*; 29%), it is significantly higher in other species (e.g., 106 infected specimens of the 130 examined in *A. longirostre*; 82%) (Tables 1 and S1). Prevalence of *Rickettsia* varied significantly between sampling localities of two tick species, *A. cajennense* (7 localities, with prevalence ranging from 0 to 48%; Fisher’s exact test, *p* = 3.10^−5^) and *A. longirostre* (14 localities, with prevalence ranging from 0 to 100%; Fisher’s exact test, *p* = 4.10^−6^) (Table S1). However, prevalence of *Rickettsia* did not vary significantly between sampling localities of the nine other tick species for which distinct geographic populations were examined (Fisher’s exact tests, all *p* > 0.05) (Table S1). Furthermore, there was no obvious difference in prevalence of *Rickettsia* between localities from peri-urban, agricultural and urban areas (Kruskal-Wallis test, *H* = 0.68, *df* = 3, *p* = 0.08).

### Diversity of *Rickettsia*

The diversity of *Rickettsia* in French Guiana ticks was examined using sequences from one to five genetic markers (*gltA*, 16S rRNA, *atpA*, *ompB* and *coxA*). Overall, the examination of this multilocus data set led to the identification of 19 distinct *Rickettsia* genotypes (FG019a–FG019s hereafter; Table 2), as detailed below.

**Table 2:** Sequence profiles of the five genes in the 19 *Rickettsia* genotypes (FG019a–FG019s) identified in this study.

Ticks Species	*Rickettsia* MLST genotypes	Genes					*n*
		*gltA*	16S rDNA	*atpA*	*ompB*	*coxA*	
**Ixodidae (hard ticks):**							
*Amblyomma cajennense* sensu stricto (Fabricius, 1787)	FG019a	a	a	a	a	a	13
*A. coelebs* Neumann, 1899	FG019b	c	b	b	c	b	3
*A. dissimile* Koch, 1884	FG019c	d	c	c	d	c	2
	FG019d	e	c	c	d	_	1
	FG019e	f	d	d	e	_	1
	FG019f	g	c	c	d	c	2
*A. geayi* Neumann, 1899	FG019c	d	c	c	d	c	1
*A. goeldii* Neumann, 1899	FG019g	d	f	e	f	d	1
	FG019h	h	e	f	g	e	1
*A. humerale* Koch, 1844	FG019i	d	c	e	i	d	2
	FG019j	i	g	g	h	_	1
*A. latepunctatum* Tonelli-Rondelli, 1939	FG019c	d	c	c	d	c	1
	FG019k	i	g	g	j	f	1
	FG019l	j	e	a	e	g	1
*A. longirostre* (Koch, 1844)	FG019m	b	a	a	b	a	4
*A. naponense* (Packard, 1869)	FG019n	k	h	h	_	_	1
*A. varium* Koch, 1844	FG019o	d	k	i	f	d	1
	FG019p	l	i	j	e	h	2
*Haemaphysalis juxtakochi* Cooley, 1946	FG019q	d	c	e	_	_	1
	FG019r	i	c	g	_	_	1
*Ixodes luciae* Senevet, 1940	FG019s	g	k	e	f	d	3

Letters a–k represent the different alleles at each gene locus. Dash indicates an absence of PCR product. *n*, number of specimens for each *Rickettsia* genotype (on the basis of multilocus typing of 44 representative tick samples).

First, *Rickettsia* sequences from the *gltA* gene were taken from a subsample of 92 infected specimens from the 12 infected species (one to 47 specimens per infected species were examined; Table 2). On the basis of DNA sequencing, 12 distinct *gltA* genotypes with 84.9–99.8% pairwise nucleotide identity were characterized from the 92 specimens examined. Six tick species of the 12 infected species harbored each only one *gltA* genotype: *A. cajennense*, *A. coelebs*, *A. longirostre*, *A. geayi*, *A. naponense* and *I. luciae*. In each of the six other tick species, two to four distinct *gltA* genotypes were found. We characterized four *gltA* genotypes from the seven sequenced *A. dissimile* specimens (Table 2).

Second, we amplified four additional bacterial markers (16S rRNA, *atpA*, *ompB* and *coxA*) from 44 representative tick samples infected by the 12 *Rickettsia gltA* genotypes (Table 2). We then obtained 10 genotypes of 16S rRNA (97.7–99.9% pairwise nucleotide identity), 10 *atpA* genotypes (85.4–99.8%), 10 *ompB* genotypes (78.1–99.8%) and eight *coxA* genotypes (86.1–99.8%). While the 16S rRNA and *atpA* gene fragments were amplified from the 44 samples, the *ompB* and *coxA* were only amplified from 41 and 38 samples, respectively (Table 2). The diversity at the 16S rRNA, *atpA*, *ompB* and *coxA* gene fragments was consistent with the results inferred from the *gltA* sequences: *Rickettsia* infections with distinct *gltA* sequences have distinct sequences at the other gene markers. No 16S rRNA, *atpA*, *ompB* and *coxA* sequence variation was observed within tick species in which only one *gltA* genotype was detected (i.e., *A. cajennense*, *A. coelebs*, *A. longirostre*, *A. geayi*, *A. naponense* and *I. luciae*). However, the combined use of these five markers allowed the distinction of additional *Rickettsia* genetic variation not detected with the single *gltA* gene sequences. Indeed, while one of the *Rickettsia* infections of *A. dissimile* and one of *A. humerale* shared the same *gltA* sequence (the *gltA* sequence type #d in Table 2), their *atpA*, *ompB* and *coxA* (but not 16S rRNA) gene sequences were different, showing that they were thus two distinct *Rickettsia* genotypes. The examination of *gltA*, 16S rRNA, *atpA*, *ompB* and *coxA* gene sequences thus led to the identification of 19 *Rickettsia* genotypes (FG019a–FG019s; Table 2). Only one of these *Rickettsia* genotypes, FG019c, was shared by several tick species (*A. dissimile*, *A. geayi* and *A. latepunctatum*). Each of the 18 other *Rickettsia* genotypes (FG019a, FG019b and FG019d–FG019s) was found in only one tick species (Table 2).

### Phylogeny of *Rickettsia*

**Figure 2:**
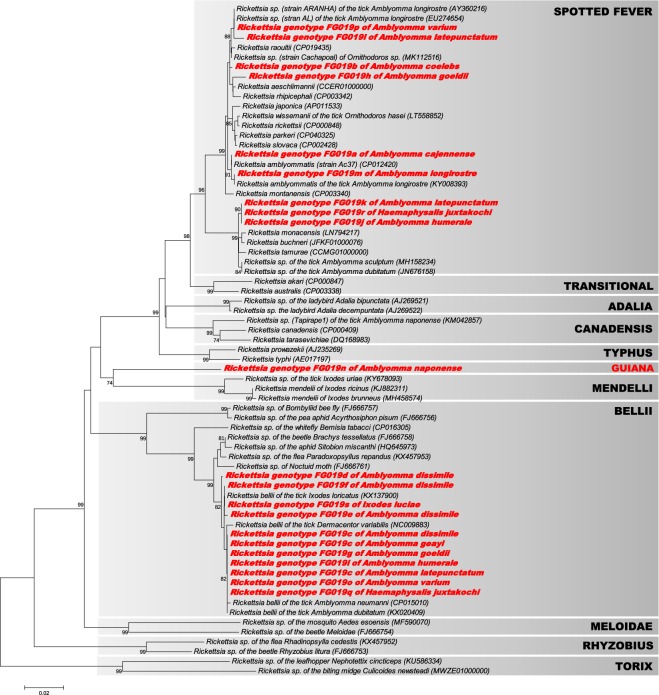
Phylogeny of *Rickettsia* constructed using maximum-likelihood (ML) estimations based on *gltA* gene sequences (589 unambiguously aligned nucleotide sites; best-fit approximation for the evolutionary model: GTR + G + I). Sequences from *Rickettsia* characterized in this study are shown in red. Only one *gltA* sequence per *Rickettsia* genotype and per tick species is shown. Sequences from representative *Rickettsia* groups, species and strains available in GenBank were also added to the analysis. The grey boxes delineate the different *Rickettsia* groups (their names are indicated in upper case), including the novel guiana group described in this study. Bacterial name, host species and GenBank accession numbers are shown on the tree. Branch numbers indicate percentage bootstrap support for major branches (1000 replicates; only bootstrap values >70% are shown). The scale bar is in units of substitution/site.

The phylogenetic relationships between the *Rickettsia* infections were first estimated using the 92 *gltA* sequences from the 12 infected tick species found in this study, as well as *gltA* sequences from representative *Rickettsia* species and strains available in GenBank (Fig. 2). The closest relatives of the *Rickettsia* found in French Guiana were also included in the analyses. No recombination events were detected for the *gltA* data set using both the RDP and GENCONV methods (all *p* > 0.23). The ML phylogenetic analysis based on the *gltA* sequences showed that the 19 *Rickettsia* genotypes (FG019a–FG019s) found in this study consisted of three distinct groups (Fig. 2):

1. Nine *Rickettsia* genotypes clustered with known members of the spotted fever group, including *R. amblyommatis* (*Rickettsia* FG019a and FG019m of *A. cajennense* and *A. longirostre*, respectively), *R. tamurae*, *R. buchneri* and *R. monacensis* (FG019j, FG019k and FG019r of *A. humerale*, *A. latepunctatum* and *H. juxtakochi*, respectively), *R. aeschlimannii* and *R. rhipicephali* (FG019h of *A. goeldii*) and *Rickettsia* sp. strains AL (FG019l and FG019p of *A. latepunctatum* and *A. varium*, respectively). The *Rickettsia* FG019b of *A. coelebs* also belonged to the spotted fever group within a cluster including *R. raoultii*, *R. aeschlimannii* and *R. rhipicephali*.
2. Nine other *Rickettsia* genotypes clustered with known members of the *R. bellii* group previously found in other tick species. These genotypes included FG019c (of *A. dissimile, A. geayi* and *A. latepunctatum*), FG019d (*A. dissimile*), FG019e (*A. dissimile*), FG019f (*A. dissimile*), FG019g (*A. goeldii*), FG019i (*A. humerale*), FG019o (*A. varium*), FG019q (*H. juxtakochi*) and FG019s (*I. luciae*).
3. The last *Rickettsia* genotype, FG019n of *A. naponense*, was genetically distant to all other known *Rickettsia* species and groups. It showed a low level of *gltA* nucleotide identity (<88%) with all other *Rickettsia* species and groups. This *Rickettsia* genotype was the single member of a novel and highly divergent group, here provisionally called the guiana group (Fig. 2). It is noteworthy that this strain is also distantly related to the *Rickettsia* sp. clone Tapirape1 (canadensis group; Fig. 2), which was previously found in *A. naponense* from Brazil^19^.

However, none of the *Rickettsia* genotypes found in this study is closely related to the single species already reported from French Guiana, *Candidatus Rickettsia* wissemanii (Fig. 2).

**Figure 3:**
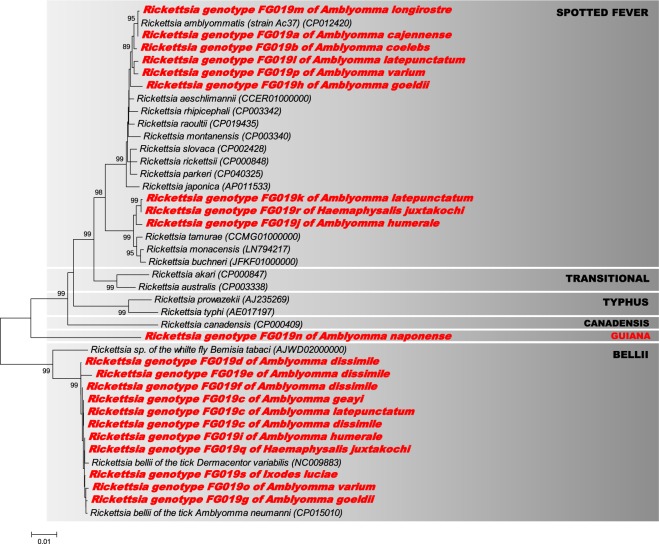
Phylogeny of *Rickettsia* constructed using maximum-likelihood (ML) estimations based on concatenated 16S rDNA, *gltA* and *atpA* sequences (1886 unambiguously aligned nucleotide sites; best-fit approximation for the evolutionary model: GTR + G + I). Sequences from *Rickettsia* characterized in this study are shown in red. Only one 16S rDNA, *gltA* and *atpA* concatenated sequence per *Rickettsia* genotype and per tick species is shown. Sequences from representative *Rickettsia* groups, species and strains available in GenBank were also added to the analysis. The grey boxes delineate the different *Rickettsia* groups (their names are indicated in upper case), including the novel guiana group described in this study. Bacterial name, host species and GenBank accession numbers are shown on the tree. Branch numbers indicate percentage bootstrap support for major branches (1000 replicates; only bootstrap values >70% are shown). The scale bar is in units of substitution/site.

A second analysis was performed to refine the intrageneric phylogeny of *Rickettsia*. For this, we used the *Rickettsia* 16S rRNA, *atpA*, *ompB* and *coxA* sequences from the 19 *Rickettsia* genotypes identified in the present work, as well as sequences of representative *Rickettsia* species and strains available in GenBank. The analysis of single and concatenated gene sequences did not detect significant recombination events in the data set using both RDP and GENCONV methods (all *p* > 0.08). When the sequences were examined separately for each gene, we obtained the same phylogenetic pattern as observed with the ML analysis based on *gltA* gene sequences with the partitioning of the 19 *Rickettsia* genotypes into the same three different groups (i.e., spotted fever, bellii and guiana) (Figs. S1–S4). Indeed, the examination of the16S rRNA and *atpA* gene sequences of *Rickettsia* FG019n genotype of *A. naponense* (neither *ompB* nor *coxA* could be amplified from this *Rickettsia* strain; see Table 2) corroborated the existence of the guiana group: the 16S rRNA, *atpA* and *gltA* single-gene phylogenies (Figs. 2, S1 and S2) and the *gltA*, 16S rRNA and *atpA* concatenated phylogeny (Fig. 3) showed that the FG019n genotype is highly divergent from all other known *Rickettsia* groups, species and strains.

Analyses of a multilocus data set (based on the 16S rRNA, *gltA* and *atpA* genes) further showed that the nine *Rickettsia* genotypes (FG019c–g, FG019i, FG019o, FG019q and FG019s) belonging to the bellii group always clustered together with the *R. bellii* strains previously found in other American tick species, such as *A. neumanni* (Argentina) and *D. variabilis* (USA) (Figs. 3 and S1–4). These nine *Rickettsia* genotypes can therefore be considered as members of the *R. bellii* species. None of these nine *Rickettsia* genotypes were 100% identical to already known *R. bellii* members.

The multilocus data s*et al*so showed that the nine *Rickettsia* genotypes (FG019a, FG019b, FG019h, FG019j–m, FG019p and FG019r) belonging the spotted fever group can be split into two subgroups:

1. The first subgroup included the *Rickettsia* FG019j (*A. humerale*), FG019k (*A. latepunctatum*) and FG019r (*H. juxtakochi*), which clustered with *R. tamurae*, *R. buchneri* and *R. monacensis* although remaining substantially divergent from them at each gene marker (Figs. 2, 3 and S1–4).
2. The second subgroup included the *Rickettsia* FG019a (*A. cajennense*), FG019m (*A. longirostre*), FG019b (*A. coelebs*), FG019h (*A. goeldii*), FG019l (*A. latepunctatum*) and FG019p (*A. varium*), which all clustered with *R. amblyommatis* on the basis of multilocus analyses (Fig. 3). However, only FG019a and FG019m consistently clustered with *R. amblyommatis* at each gene marker (Figs. 2 and S1–4), showing that these two genotypes can be considered as members of the *R. amblyommatis* species. None of these two *Rickettsia* genotypes were 100% identical to already known *R. amblyommatis* members, however. The four other *Rickettsia* genotypes (FG019b, FG019h, FG019l and FG019p) cannot be classified into specific species due to a lack of consensus between the phylogenetic trees (Figs. 2, 3 and S1–4). Indeed, while the 16S rDNA genotype of FG019b is more closely related to *R. amblyommatis* (Fig. S1), its *atpA* genotype is more closely related to *R. montanensis* (Fig. S2). Although these last four *Rickettsia* genotypes may each represent a novel species, additional gene sequencing is required to determine their precise phylogenetic proximity with other members of the spotted fever group.

## Discussion

We found here that *Rickettsia* infections are common in French Guiana ticks, a pattern also observed among ticks of other South American regions^7,10–13,20^. The incidence of infection varied between tick species of French Guiana: 12 of the 22 tick species examined, including *Amblyomma*, *Haemaphysalis* and *Ixodes* species, harbored *Rickettsia*, and when present, prevalence ranged from 10 to 100%, with significant variations between sampling localities of some species. We further found that these *Rickettsia* infections are remarkably diverse. Multilocus strain typing revealed the presence of 19 *Rickettsia* genotypes of different phylogenetic origins. Of these 19 genotypes, 18 were found each in only one tick species. Remarkably, more than one *Rickettsia* genotype was found within half of the infected tick species, meaning that this intraspecific variation of infection is common in tick species of French Guiana. The single *Rickettsia* species known from this region, *Candidatus* Rickettsia wissemanii^9^, was not detected in the present study. Altogether, this means that at least 20 different *Rickettsia* genotypes are circulating in ticks in French Guiana.

None of the 19 *Rickettsia* genotypes we identified in French Guiana had been documented before this study. Multilocus typing showed that 11 of these *Rickettsia* genotypes can be assigned to two validated *Rickettsia* species, namely *R. amblyommatis* (two genotypes) and *R. bellii* (nine genotypes). These two *Rickettsia* species are widely present among Central and South American ticks, each infecting more than 10 species^7,11–13,21–26^. Their presence in French Guiana was therefore expected, but the observation of novel genotypes indicates the presence of important geographic variability: *R. amblyommatis* and *R. bellii* have probably radiated within their respective regions, including French Guiana, into different genotypes. Overall, this confirms that *R. amblyommatis* and *R. bellii* have the widest host range and the broadest geographic distribution among all *Rickettsia* species reported from South America, as suggested in early studies^7,25^. Besides the *R. amblyommatis* and *R. bellii* genotypes, the eight other *Rickettsia* genotypes are rarer, since they are apparently endemic to French Guiana and cannot be assigned to formerly validated species. While a few genotypes remain unclassified within the spotted fever group, we described one novel *Rickettsia* genotype, which belongs to a novel and divergent group, the guiana group. It is noteworthy that the guiana group has an intermediate phylogenetic position between the spotted fever and bellii *Rickettsia* groups, since it is more related on the basis of its *gltA* sequence to the rare species *R. mendelii*, which was found only in Europe^27,28^.

Most of human pathogenic *Rickettsia* species are vectored by hard ticks^4,29^. This leads to the question of the pathogenicity of the 19 *Rickettsia* genotypes we found in French Guiana and the associated health risk. Since none of the 19 *Rickettsia* genotypes was previously described before this study, no evidence of their pathogenicity currently exists, even for those belonging to validated *Rickettsia* species. Indeed, although *R. amblyommatis* and *R. bellii* are commonly found in the ticks of French Guiana, and more broadly in American ticks^7,11–13,21–26^, they have never been found in vertebrate hosts, suggesting that they are nonpathogenicspecies. Interestingly, while the Cayenne tick *A. cajennense* is one of the most common ticks found in French Guiana, blood-feeding on many different hosts, including humans^8,30,31^, *R. amblyommatis* (infecting here 25% of the *A. cajennense* specimens examined) were never detected in humans or animals: while French Guiana is an outermost region of the European Union, with technical and financial resources that close to European countries, no case was notified to date. Another intriguing point is the apparent specificity of *Rickettsia* genotypes to tick species: 18 out of 19 *Rickettsia* genotypes were detected in only a single tick species. Even generalist tick species, such as *A. cajennense* and *A. dissimile*, feeding on (and sharing) a variety of vertebrate hosts^8,30,31^, did not share the same *Rickettsia* genotypes. These observations may indicate that at least some of the *Rickettsia* genotypes in French Guiana are present in ticks but not in vertebrate hosts.

The persistence means of the 19 *Rickettsia* genotypes remain unknown in French Guiana. As pointed out in a recent study^32^, the current view in rickettsiology has a strong anthropocentric bias and tends to describe all novel *Rickettsia* species as pathogenic forms. However, most of the novel *Rickettsia* species or strains discovered in recent years are also found exclusively in arthropods and never in vertebrates^1–3,33,34^. In ticks, as for many other arthropods, some *Rickettsia* are maternally inherited endosymbionts with poorly known effects on tick biology. This is the case for *R. buchneri* in the black-legged tick *I. scapularis*^35^, *R. peacockii* in the American dog tick *D. variabilis*^36^, and *R. vini* in the tree-hole tick *I. arboricola*^16,37^. These nonpathogenic *Rickettsia* may interact with a variety of tick-borne pathogens^34^, including *Anaplasma marginale*^38^, *Borrelia burgdorferi*^39^ and also other *Rickettsia*^40,41^. Indeed, the endosymbiont *R. peacockii* may possibly hamper the multiplication of the spotted fever agent, *R. rickettsii*^40^, and may also block transovarial transmission colonization of *R. rickettsii*, *R. montana* and *R. rhipicephali*^40,41^. In French Guiana, further studies are needed to test this hypothesis of endosymbiosis by observing transstadial and transovarial transmission in ticks.

To conclude, this study revealed substantial diversity of *Rickettsia*, including novel genotypes, specie and group, in ticks in French Guiana. This underlines the need to better document *Rickettsia* diversity in diverse regions, and more especially in remote regions. A recent meta-analysis suggests that more than 20% of terrestrial arthropods may be infected by *Rickettsia*, with ticks hosting most of this bacterial diversity^29^, as observed in this study. In many arthropods other than ticks, *Rickettsia* are nonpathogenic, undergo exclusive maternal transmission to offspring, and may function as both a mutualist and reproductive manipulator^2,3,42,43^. Overall, adaptations of *Rickettsia* to this diversity of hosts encompass an array of parasitic, but also mutualistic, interactions^1–3^. In French Guiana, the effect of the19 *Rickettsia* genotypes on human and animal health as well as on tick physiology and reproduction remains to be elucidated.

## Materials and Methods

### Tick collection

A collection of 819 specimens from 22 tick species, collected in 38 sample sites of French Guiana in 2016 and 2017, was used (Tables 1 and S1). Questing ticks were collected from the vegetation using a drag-flag method over sites covering three types of ecological conditions (periurban, agricultural and natural). Ticks were also directly collected in nests or on hosts (including humans, four domestic animal species and wild animal species; see Table S1). All ticks were stored in 75% ethanol until examination. For each tick specimen, species were formally identified through morphological examination (using dichotomous keys^30,44^) and DNA sequencing in a previous study^8^.

### Detection of *Rickettsia*

To avoid external bacterial contaminants, ticks were processed with commercial bleach diluted at 1% for 30 s and then rinsed for 1 min in three successive baths of DNA-free water following a published protocol^45^. For each tick specimen, total DNA was further extracted from whole body using a genomic DNA extraction kit according to the manufacturer’s instructions (DNeasy Blood & Tissue extraction kit, Qiagen). The presence of *Rickettsia* within each DNA template was investigated through high-throughput 16S rDNA sequencing. To this aim, a 251-bp portion of the V4 variable region of the bacterial 16S rDNA was amplified from whole-body DNA samples using the universal forward and reverse primers listed in Table S4. Each PCR product from individual samples was tagged with a unique 35-base barcode using the Nextera Index Kit (Illumina, San Diego, CA, USA). PCR amplifications were performed in duplicates for each sample. PCR reactions were conducted using a Multiplex PCR Kit (Qiagen). Amplified bacterial 16S rDNA products were purified and sequenced on an Illumina MiSeq platform (GenSeq, Montpellier University) and 250-bp end sequence reads were obtained. All bioinformatic analyses were conducted using the pipeline Frogs (https://github.com/geraldinepascal/FROGS) as follows^46^: primers were removed from paired-end sequences with Cutadapt^47^, and these sequences were merged into contigs with FLASH^48^ before filtering by length (251 bp ± 10 bp). Chimaeras were removed with VSEARCH^49^, then sequences were clustered using SWARM^50^. We obtained an average number of 29,206 bacterial 16S rDNA reads per tick specimen. Sequences with 97% similarity were clustered together and identified as an operational taxonomic unit (OTU). Each representative OTU sequence was aligned and taxonomically assigned using the Silva database (https://www.arb-silva.de/). To eliminate the possibility of contamination, we included four mock DNA extractions under identical conditions using water, buffers and kits utilized for the experimental samples followed by Illumina Miseq analysis of 16S rDNA reads. The negative controls provided only a handful of reads that did not correspond to the bacterial genera found in the tick samples.

### Molecular typing of *Rickettsia*

A random subset of DNA templates for which *Rickettsia* reads were obtained through high-throughput 16S rDNA sequencing were used for *Rickettsia* multilocus typing. These *Rickettsia* infections were genotyped using independent PCR assays based on *gltA*, *coxA*, *ompB*, *atpA* and 16S rRNA, using semi-nested or nested PCR assays (Table S2). To prevent possible contamination, different parts of this process were physically separated from one another, in entirely separate rooms. All amplicons were also sequenced to control for false-positive amplifications. Gene features, primers and PCR conditions are detailed in Table S2.

Seminested and nested PCR amplifications were performed as follows: the first PCR run with the external primers was performed in a 10-μL volume containing approximately 20 ng of genomic DNA, 3 mM of each dNTP (Thermo Scientific), 8 mM of MgCl_2_ (Roche Diagnostics), 3 μM of each primer, 1 μL of 10× PCR buffer (Roche Diagnostics) and 0.5 U of Taq DNA polymerase (Roche Diagnostics). A 1-μL aliquot of the PCR product from the first reaction was then used as a template for the second round of amplification. The second PCR was performed in a total volume of 25 μL and contained 8 mM of each dNTP (Thermo Scientific), 10 mM of MgCl_2_ (ThermoScientific), 7.5 μM of each of the internal primers, 2.5 μL of 10× PCR buffer (Thermo Scientific) and 1.25 U of Taq DNA polymerase (Thermo Scientific). All PCR amplifications were performed under the following conditions: initial denaturation at 93 °C for 3 min, 35 cycles of denaturation (93 °C, 30 s), annealing (Tm = 52–56 °C, depending on primers, 30 s), extension (72 °C, 1 min), and a final extension at 72 °C for 5 min. Known positive and negative individuals were used as controls in each PCR assay. All PCR products were visualized through electrophoresis in a 1.5% agarose gel. Positive PCR products were purified and sequenced in both directions (EUROFINS). The chromatograms were manually inspected and cleaned with CHROMAS LITE (http://www.technelysium.com.au/chromas_lite.html) and sequence alignments were done using CLUSTALW^51^, both implemented in MEGA7. Genotype naming (ie,) was based on the following rationale: the genotype FG019a means French Guiana 2019 genotype a. Novel nucleotide sequences were deposited in the GenBank nucleotide database (Accession numbers: *gltA*, MT009163-MT009163; 16S rRNA, MT006105-MT006125; *coxA*, MT009148-MT009162; *ompB*, MT009184-MT009201; *atpA*, MT009127-MT009147).

### Molecular phylogenetics

The GBLOCKS^52^ program with default parameters was used to remove poorly aligned positions and to obtain unambiguous sequence alignments. All sequence alignments were also checked for putative recombinant regions using the RDP3 computer analysis package^53^. Given a set of aligned nucleotide sequences, RDP3 can rapidly analyze these with a range of powerful nonparametric recombination detection methods, including the GENECON^54^ and RDP^55^. Phylogenetic relationships were evaluated between *Rickettsia* strains using *gltA*, *coxA*, *ompB*, *atpA* and 16S rRNA gene sequences. The evolutionary models most closely fitting the sequence data were determined using Akaike information criterion with the MEGA7 program^56^. Phylogenetic analyses were based on maximum likelihood (ML) analyses. A ML heuristic search, using a starting tree obtained by neighbor-joining, was conducted, and clade robustness was further assessed by bootstrap analysis using 1000 replicates in MEGA7^56^.

### Ethics approval

The use of the genetic resources was declared to the French Ministry of the Environment under reference TREL19028117S/156 and #150401230100, in compliance with the Access and Benefit Sharing procedure implemented by the Loi pour la Reconquête de la Biodiversité. The capture of ticks in the Grand Connétable protected area was authorized by the Prefecture of French Guiana by prefectoral decree R03-2016-09-23-003. All animals were handled in strict accordance with good animal practices as defined by the French code of practice for the care and use of animals for scientific purposes, established by articles R214-87 to R214-137 of the French rural code.

## Supplementary information

Supplementary dataset.

## Data Availability

Nucleotide sequences of *Rickettsia* were deposited in the GenBank nucleotide database (Accession numbers: 16S rRNA: [MT006105-MT006125]; *gltA*: [MT009163-MT009183]; *ompB*: [MT009184-MT009201]; *atpA*: [MT009127-MT009147]; *coxA*: [MT009148-MT0091462]).

## References

[1] Weinert, L. A. The diversity and phylogeny of *Rickettsia*. *Parasite Diversity and Diversification: Evolutionary Ecology Meets Phylogenetics* (2015)

[2] Weinert LA, Werren JH, Aebi A, Stone GN, Jiggins FM (2009). Evolution and diversity of *Rickettsia* bacteria. BMC Biol..

[3] Perlman SJ, Hunter MS, Zchori-Fein E (2006). The emerging diversity of *Rickettsia*. Proc. Biol. Sci..

[4] Parola P, Paddock CD, Raoult D (2005). Tick-borne rickettsioses around the world: emerging diseases challenging old concepts. Clin. Microbiol. Rev..

[5] Raoult D, Roux V (1997). Rickettsioses as paradigms of new or emerging infectious diseases. Clin. Microbiol. Rev..

[6] Parola P (2013). Update on tick-borne rickettsioses around the world: a geographic approach. Clin. Microbiol. Rev..

[7] Labruna MB (2009). Ecology of *Rickettsia* in South America. Ann. N. Y. Acad. Sci..

[8] Binetruy F, Chevillon C, de Thoisy B, Garnier S, Duron O (2019). Survey of ticks in French Guiana. Ticks and Tick-borne. Dis..

[9] Tahir D (2016). New *Rickettsia* species in soft ticks Ornithodoros hasei collected from bats in French Guiana. Ticks Tick-borne Dis..

[10] Guedes E, Leite RC, Pacheco RC, Silveira I, Labruna MB (2011). *Rickettsia* species infecting *Amblyomma* ticks from an area endemic for Brazilian spotted fever in Brazil. Rev. Bras. Parasitol. Vet..

[11] Amoêdo-Lima M (2018). Ticks and tick-associated spotted fever group *Rickettsia* from birds in the Southwestern Brazilian Amazon. Rev. Colombiana de. Cienc. Pecuarias.

[12] Ogrzewalska M, Literak I, Martins TF, Labruna MB (2014). *Rickettsia*l infections in ticks from wild birds in Paraguay. Ticks Tick. Borne Dis..

[13] McIntosh D (2015). Detection of *Rickettsia* bellii and *Rickettsia* amblyommii in *Amblyomma* longirostre (Acari: Ixodidae) from Bahia state, Northeast Brazil. Braz. J. Microbiol..

[14] Ogrzewalska M, Uezu A, Labruna MB (2010). Ticks (Acari: Ixodidae) infesting wild birds in the eastern Amazon, northern Brazil, with notes on *rickettsia*l infection in ticks. Parasitol. Res..

[15] Lalzar I, Friedmann Y, Gottlieb Y (2014). Tissue tropism and vertical transmission of Coxiella in Rhipicephalus sanguineus and Rhipicephalus turanicus ticks. Environ. Microbiol..

[16] Duron O (2017). Evolutionary changes in symbiont community structure in ticks. Mol. Ecol..

[17] Duron O (2018). Tick-bacteria mutualism depends on b vitamin synthesis pathways. Curr. Biol..

[18] Clayton KA, Gall CA, Mason KL, Scoles GA, Brayton KA (2015). The characterization and manipulation of the bacterial microbiome of the Rocky Mountain wood tick, Dermacentor andersoni. Parasit. Vectors.

[19] Soares HS (2015). Ticks and *rickettsia*l infection in the wildlife of two regions of the Brazilian Amazon. Exp. Appl. Acarol..

[20] Ogrzewalska M, Literak I, Cardenas-Callirgos JM, Capek M, Labruna MB (2012). *Rickettsia* bellii in ticks *Amblyomma* varium Koch, 1844, from birds in Peru. Ticks Tick. Borne Dis..

[21] Bermúdez CSE, Troyo A (2018). A review of the genus *Rickettsia* in Central America. Res. Rep. Trop. Med..

[22] Lopes, M. G. *et al*. Ticks and rickettsiae from wildlife in Belize, Central America. *Parasit Vectors* **9** (2016). 10.1186/s13071-016-1348-1 PMC4736261 26831147

[23] Polsomboon S (2017). Molecular detection and identification of *Rickettsia* species in ticks (acari: ixodidae) collected from Belize, Central America. J. Med. Entomol..

[24] Muñoz-Leal S, Marcili A, Fuentes-Castillo D, Ayala M, Labruna MB (2019). A relapsing fever Borrelia and spotted fever *Rickettsia* in ticks from an Andean valley, central Chile. Exp. Appl. Acarol..

[25] Krawczak FS, Labruna MB, Hecht JA, Paddock CD, Karpathy SE (2018). Genotypic characterization of *Rickettsia* bellii reveals distinct lineages in the United States and South America. Biomed. Res. Int..

[26] Ogrzewalska M, Pacheco RC, Uezu A, Ferreira F, Labruna MB (2008). Ticks (Acari: Ixodidae) infesting wild birds in an Atlantic forest area in the state of São Paulo, Brazil, with isolation of *Rickettsia* from the tick *Amblyomma* longirostre. J. Med. Entomol..

[27] Hajduskova E (2016). ‘Candidatus *Rickettsia* mendelii’, a novel basal group *rickettsia* detected in Ixodes ricinus ticks in the Czech Republic. Ticks Tick. Borne Dis..

[28] Stańczak J, Biernat B, Racewicz M, Zalewska M, Matyjasek A (2018). Prevalence of different *Rickettsia* spp. in Ixodes ricinus and Dermacentor reticulatus ticks (Acari: Ixodidae) in north-eastern Poland. Ticks Tick. Borne Dis..

[29] Weinert LA, Araujo-Jnr EV, Ahmed MZ, Welch JJ (2015). The incidence of bacterial endosymbionts in terrestrial arthropods. Proc. Biol. Sci..

[30] Floch H, Fauran P (1958). Ixodides de la Guyane et des Antilles Françaises. Publ. Inst. Pasteur Guyane Fr. Inini.

[31] Nava, S., Venzal, J. M., Acuña, D. G., Martins, T. F. & Guglielmone, A. A. *Ticks of the Southern Cone of America: Diagnosis, Distribution, and Hosts with Taxonomy, Ecology and Sanitary Importance*. (Academic Press, 2017).

[32] Labruna MB, Walker DH (2014). *Rickettsia* felis and changing paradigms about pathogenic rickettsiae. Emerg. Infect. Dis..

[33] Darby AC, Cho N-H, Fuxelius H-H, Westberg J, Andersson SGE (2007). Intracellular pathogens go extreme: genome evolution in the rickettsiales. Trends genetics: TIG.

[34] Bonnet, S. I., Binetruy, F., Hernández-Jarguín, A. M. & Duron, O. The tick microbiome: why non-pathogenic microorganisms matter in tick biology and pathogen transmission. *Front Cell Infect Microbiol* **7** (2017). 10.3389/fcimb.2017.00236 PMC5462901 28642842

[35] Kurtti TJ (2015). *Rickettsia* buchneri sp. nov., a rickettsial endosymbiont of the blacklegged tick *Ixodes scapularis*. Int. J. Syst. Evol. Microbiol..

[36] Felsheim RF, Kurtti TJ, Munderloh UG (2009). Genome sequence of the endosymbiont *Rickettsia peacockii* and comparison with virulent *Rickettsia rickettsii*: Identification of virulence factors. PLOS ONE.

[37] Novakova, M., Costa, F. B., Krause, F., Literak, I. & Labruna, M. B. *Rickettsia* vini n. sp. (Rickettsiaceae) infecting the tick Ixodes arboricola (Acari: Ixodidae). *Parasit Vectors* **9** (2016). 10.1186/s13071-016-1742-8 PMC5000463 27565956

[38] Gall CA (2016). The bacterial microbiome of Dermacentor andersoni ticks influences pathogen susceptibility. ISME J..

[39] Steiner FE (2008). Infection and co-infection rates of Anaplasma phagocytophilum variants, Babesia spp., *Borrelia burgdorferi*, and the rickettsial endosymbiont in *Ixodes scapularis* (Acari: Ixodidae) from sites in Indiana, Maine, Pennsylvania, and Wisconsin. J. Med. Entomol..

[40] Burgdorfer, W., Hayes, S. F & Mavros, A. J. Nonpathogenic rickettsiae in Dermacentor andersoni: a limiting factor for the distribution of *Rickettsia* rickettsii. *Rickettsiae and rickettsial diseases* (1980).

[41] Macaluso KR, Sonenshine DE, Ceraul SM, Azad AF (2002). Rickettsial infection in *Dermacentor variabilis* (Acari: Ixodidae) inhibits transovarial transmission of a second *Rickettsia*. J. Med. Entomol..

[42] Behar A, McCormick LJ, Perlman SJ (2010). *Rickettsia* felis infection in a common household insect pest, Liposcelis bostrychophila (*Psocoptera: Liposcelidae*). Appl. Environ. Microbiol..

[43] Gottlieb Y (2006). Identification and localization of a *Rickettsia* sp. in Bemisia tabaci (Homoptera: Aleyrodidae). Appl. Environ. Microbiol..

[44] Jones, E. K., Clifford, C. M., Keirans, J. E. & Kohls, G. M. Ticks of Venezuela (Acarina: Ixodoidea) with a key to the species of *Amblyomma* in the Western Hemisphere. Brigham Young University Science Bulletin, Biological Series **17** (1972).

[45] Binetruy F, Dupraz M, Buysse M, Duron O (2019). Surface sterilization methods impact measures of internal microbial diversity in ticks. Parasites Vectors.

[46] Escudié F (2018). FROGS: Find, Rapidly, OTUs with Galaxy Solution. Bioinforma..

[47] Martin M (2011). Cutadapt removes adapter sequences from high-throughput sequencing reads. EMBnet.journal.

[48] Magoč T, Salzberg SL (2011). FLASH: fast length adjustment of short reads to improve genome assemblies. Bioinforma..

[49] Rognes T, Flouri T, Nichols B, Quince C, Mahé F (2016). VSEARCH: a versatile open source tool for metagenomics. PeerJ.

[50] Mahé F, Rognes T, Quince C, de Vargas C, Dunthorn M (2014). Swarm: robust and fast clustering method for amplicon-based studies. PeerJ.

[51] Thompson, J. D., Gibson, T. J. & Higgins, D. G. Multiple sequence alignment using ClustalW and ClustalX. *Curr Protoc Bioinformatics* Chapter 2, Unit 2.3 (2002). 10.1002/0471250953.bi0203s00 18792934

[52] Castresana J (2000). Selection of conserved blocks from multiple alignments for their use in phylogenetic analysis. Mol. Biol. Evol..

[53] Martin DP (2010). RDP3: a flexible and fast computer program for analyzing recombination. Bioinforma..

[54] Sawyer S (1989). Statistical tests for detecting gene conversion. Mol. Biol. Evol..

[55] Martin D, Rybicki E (2000). RDP: detection of recombination amongst aligned sequences. Bioinforma..

[56] Kumar S, Stecher G, Tamura K (2016). MEGA7: Molecular Evolutionary Genetics Analysis Version 7.0 for bigger datasets. Mol. Biol. Evol..

