# Supplementary material for: Novel Rickettsia genotypes in ticks in French Guiana, South America
Source: Sci Rep. 2020 Feb 13;10:2537. doi: 10.1038/s41598-020-59488-0 (PMC7018960; doi:10.1038/s41598-020-59488-0)
Supplement: Supplementary file 1 — Supplementary dataset. [file 41598_2020_59488_MOESM1_ESM.pdf]

SUPPLEMENTARY MATERIALS

Novel *Rickettsia* genotypes in ticks in French Guiana, South America

Florian Binetruy, Marie Buysse, Roxanne Barosi and Olivier Duron

Table S1. Detailed list of tick species included in the analysis, with details on their origin, the population sample size (*n*), and the prevalence of *Rickettsia*.

Ticks Species	Locality (# on Figure 1)	Coordinates	Ecological habitat	Collected on host/habitat	Stage	*n* examined	*n Rickettsia*-positive (%)
Ixodidae (hard ticks):							
1 *Amblyomma cajennense* sensus stricto (Fabricius, 1787)	Sinnamary, Piste Saint-Elie, 2016 (#6)	5°22'3"N 53°01'3"W	Degraded forest	Vegetation	Nymphs	5	2 (40%)
	Kourou, CSG, 2016 (#11)	5°11'3"N 52°46'5"W	Old-growth forests	Vegetation	Nymphs and adults	72	11 (15%)
	Kourou, Montagne des Singes, 2016 (#13)	5°04'2"N 52°41'5"W	Old-growth forests	Vegetation	Nymphs	12	0 (0%)
	Matoury, Piste de la Mirande, 2016 (#1)	4°51'5"N 52°20'3"W	Degraded forest	Vegetation	Nymphs and adults	64	31 (48%)
	Matoury, Piste de la Mirande, 2017 (#43)	4°51'5"N 52°20'3"W	Degraded forest	Vegetation	Adults	150	33 (22%)
	Kaw, Montagne de Kaw, 2016 (#2)	4°33'6"N 52°11'W	Old-growth forests	Vegetation	Nymphs	28	9 (32%)
	Petit-Saut, Montagne Plomb, 2016 (#7)	5°04'2"N 52°58'5"W	Old-growth forests	Vegetation	Nymphs	20	2 (10%)
2 *A. calcaratum* Neumann, 1899	Kourou, Montagne des Singes, 2016 (#22)	5°04'2"N 52°41'5"W	Old-growth forests	White-bearded Manakin (*Manacus manacus*)	Larva	1	0 (0%)
3 *A. coelebs* Neumann, 1899	Petit-Saut, Montagne Plomb, 2016 (#7)	5°04'2"N 52°58'5"W	Old-growth forests	Vegetation	Nymphs	11	4 (36%)
	Kaw, Montagne de Kaw, 2016 (#2)	4°33'6"N 52°11'W	Old-growth forests	Vegetation	Nymph	1	0 (0%)
	Sinnamary, Piste Saint-Elie, 2016 (#6)	5°22'3"N 53°01'3"W	Degraded forest	Vegetation	Nymph	1	0 (0%)
	Sinnamary, Crique Verte, 2016 (#34)	5°21'2"N 52°59'5"W	Old-growth forests	Vegetation	Nymph	1	0 (0%)
4 *A. dissimile* Koch, 1884	Awala-Yalimapo, Ayawande, 2016 (#36)	5°40'1"N 53°56'6"W	Peri-urban	Cane toad (*Rhinella marina*)	Nymphs and adults	4	1 (25%)
	Rémire-Montjoly, 2017 (#44)	4°54'4"N 52°16'05"W	Peri-urban	Green Iguana (*Iguana iguana*)	Nymphs and adults	14	11 (79%)
	Rémire-Montjoly, 2018 (#45)	4°53'6"N 52°15'5"W	Peri-urban	Green Iguana (*Iguana iguana*)	Adults	3	3 (100%)
	Matoury, Piste de la Mirande, 2016 (#1)	4°51'5"N 52°20'3"W	Degraded forest	Vegetation	Nymph	1	0 (0%)
	Talhuen, Village, 2016 (#17)	3°23'1"N 54°2'2"W	Peri-urban	Vegetation	Nymphs	2	1 (50%)
5 *A. geayi* Neumann, 1899	Cayenne, Montagne Tigre, 2017 (#40)	4°54'5"N 52°18'0"W	Peri-urban	Vegetation	Larvae	4	2 (50%)
	Rémire-Montjoly, Sentier du Rorota, 2014 (#19)	4°52'3"N 52°15'1"W	Degraded forest	Crimson-hooded Manakin (*Pipra aureola*) and Woodcreeper (*Glyphoryncus spirurus*)	Larvae	3	2 (67%)
	Rémire-Montjoly, Sentier du Rorota, 2016 (#8)	4°53'N 52°15'5"W	Degraded forest	Crimson-hooded Manakin (*Pipra aureola*) and Barred Antshrike (*Thamnophilus doliatus*)	Larvae	5	2 (40%)

		Rémire-Montjoly, Vidal, 2016 (#32)	4°51'5''N 52°15'2''W	Degraded forest	Sloth (*Bradypus tridactylus*)	Adults	2	2	(100%)
		Saint-Laurent-du-Maroni, Piste Voltaire, 2013 (#21)	5°02'5''N 54°04'3''W	Old-growth forests	White-bearded Manakin (*Manacus manacus*) and White-necked Thrush (*Turdus albicollis*)	Larvae	2	2	(100%)
6	*A. goeldii* Neumann, 1899	Régina, N1 road PK63, 2017 (#38)	4°28'5''N 52°21'4''W	Degraded forest	Tamandua (*Tamandua tetradactyla*)	Adults	5	4	(80%)
7	*A. humerale* Koch, 1844	Kourou, Montagne des Singes, 2016 (#13)	5°04'2''N 52°41'5''W	Old-growth forests	Human	Nymph	1	0	(0%)
		Station des Nouragues, 2017 (#47)	4°05'2''N 52°40'5''W	Old-growth forests	Vegetation	Adults	5	1	(20%)
		Matoury, RN, 2016 (#15)	4°47'3''N 52°23'2''W	Degraded forest	Gray Four-eyed Opossum (*Philander opossum*)	Nymphs	3	3	(100%)
		Mana, Trinité, 2017 (#42)	4°50'4''N 53°36'1''W	Old-growth forests	Human	Adult	1	1	(100%)
8	*A. latepunctatum* Tonelli-Rondelli, 1939	Saint-Georges, Crique Gabaret 2016 (#33)	3°54'2''N 51°47'4''W	Pasture	Vegetation	Nymph	1	1	(100%)
		Petit-Saut, Montagne Plomb, 2016 (#7)	5°04'2''N 52°58'5''W	Old-growth forests	Vegetation	Nymphs	3	3	(100%)
9	*A. longirostre* (Koch, 1844)	Montsinéry, Bagne des Annamites, 2014 (#23)	4°50'1''N 52°31'W	Degraded forest	McConnell's flycatcher (*Mionectes macconnelli*)	Larvae	2	0	(0%)
		Macouria, lotissement Maillard, 2012 (#25)	4°58'N 52°27'W	Degraded forest	Woodcreeper (*Glyphoryncus spirurus*) and Blue-backed Manakin (*Chiroxiphia pareola*)	Larvae	18	13	(72%)
		Macouria, lotissement Maillard, 2013 (#27)	4°58'N 52°27'W	Degraded forest	Woodcreeper (*Glyphoryncus spirurus*)	Larvae	3	3	(100%)
		Macouria, lotissement Maillard, 2014 (#28)	4°58'N 52°27'W	Degraded forest	Blue-backed Manakin (*Chiroxiphia pareola*) and Crimson-hooded Manakin (*Pipra aureola*)	Larvae and nymph	6	6	(100%)
		Kourou, Montagne des Pères, 2014, (#24)	5°06'5''N 52°38'5''W	Old-growth forests	Blue-backed Manakin (*Chiroxiphia pareola*) and Crimson-hooded Manakin (*Pipra aureola*)	Larvae and nymph	12	10	(83%)
		Kourou, Montagne des Singes, 2014 (#22)	5°04'3''N 52°41'5''W	Old-growth forests	Woodcreeper (*Glyphoryncus spirurus*), White-bearded Manakin (*Manacus manacus*), White-flanked Antwren (*Myrmotherula axillaris*) and Golden-headed Manakin (*Pipra erythrocephala*)	Larvae and nymph	5	2	(40%)
		Rémire-Montjoly, Sentier du Rorota, 2014 (#19)	4°52'3''N 52°15'1''W	Degraded forest	Woodcreeper (*Glyphoryncus spirurus*), Blue-backed Manakin (*Chiroxiphia pareola*) and Crimson-hooded Manakin (*Pipra aureola*)	Larvae	29	29	(100%)
		Rémire-Montjoly, Sentier du Rorota, 2016 (#8)	4°53'N 52°15'5''W	Degraded forest	Crimson-hooded Manakin (*Pipra aureola*), Barred Antshrike (*Thamnophilus doliatus*), Buff-throated Saltator (*Saltator maximus*), Silver-beaked Tanager (*Ramphocelus carbo*), Yellow-olive Flatbill (*Tolmomyias sulphurescens*), Straight-billed Woodcreeper (*Dendroplex picus*) and Golden-spangled Piculet (*Picumnus exilis*)	Larvae	18	15	(83%)
		Matoury, Mont Paramana, 2014 (#30)	4°48'5''N 52°19'6''W	Degraded forest	Crimson-hooded Manakin (*Pipra aureola*) and White-bearded Manakin (*Manacus manacus*)	Larvae and nymph	14	11	(79%)
		Saint-Laurent-du-Maroni, Piste Voltaire, 2013 (#21)	5°02'5''N 54°04'3''W	Old-growth forests	Grey-breasted Sabrewing (*Campylopterus largipennis*)	Larva	1	1	(100%)
		Matoury, PK7, 2012 (#26)	4°52'4''N 52°20'1''W	Peri-urban	White-bearded Manakin (*Manacus manacus*)	Larva	1	0	(0%)
		Montsinéry, Chemin de Risquetout, 2014 (#29)	4°54'4''N 52°35'4''W	Old-growth forests	McConnell's flycatcher (*Mionectes macconnelli*) and White-necked Thrush (*Turdus albicollis*)	Larva and nymph	2	0	(0%)
		Saint-Laurent-du-Maroni, Saint-Jean, 2013 (#20)	5°24'N 54°04'5''W	Degraded forest	McConnell's flycatcher (*Mionectes macconnelli*) and Woodcreeper (*Glyphoryncus spirurus*)	Larvae	2	0	(0%)
		Rémire-Montjoly, Vidal, 2013 (#31)	4°51'5''N 52°15'2''W	Degraded forest	Woodcreeper (*Glyphoryncus spirurus*), Blue-backed Manakin (*Chiroxiphia pareola*) and Crimson-hooded Manakin (*Pipra aureola*)	Larvae and nymph	17	16	(94%)
10	*A. naponense* (Packard, 1869)	Kaw, Montagne de Kaw, 2016 (#2)	4°33'6''N 52°11'W	Old-growth forests	Vegetation	Nymphs	3	0	(0%)
		Saint-Georges, crique Gabaret, 2016 (#33)	3°54'2''N 51°47'4''W	Degraded forest	Vegetation	Adult	1	0	(0%)

		Mana, Trinité, 2017 (#42)	4°50'4"N 53°36'1"W	Old-growth forests	Human	Adult	1	1	(100%)
11	*A. oblongoguttatum* Koch, 1844	Sinnamary, Piste Saint-Elie, 2016 (#6)	5°22'3"N 53°01'3"W	Degraded forest	Vegetation	Nymph	24	0	(0%)
		Kourou, CSG, 2016 (#11)	5°11'3"N 52°46'5"W	Old-growth forests	Human	Adults	70	0	(0%)
		Petit-Saut, Montagne Plomb, 2016 (#7)	5°04'2"N 52°58'5"W	Old-growth forests	Vegetation	Nymph	1	0	(0%)
12	*A. pacae* Aragão, 1911	Saint-Georges, crique Gabaret, 2016 (#31)	3°54'2"N 51°47'4"W	Degraded forest	Vegetation	Larvae	6	0	(0%)
		Kourou, CSG, 2016 (#11)	5°11'3"N 52°46'5"W	Old-growth forests	Human	Nymph	1	0	(0%)
13	*A. romitii* Tonelli-Rondelli, 1939	Macouria, Tonate, 2017 (#41)	5°0'5"N 52°28'3"W	Peri-urban	Capybara (*Hydrochoerus hydrochaeris*)	Adults	2	0	(0%)
14	*A. rotundatum* Koch, 1844	Iracoubo, Counamie, 2017 (#39)	5°26'4"N 53°09'2"W	Degraded forest	Red-footed tortoise (*Chelonoidis carbonaria*)	Adult	1	0	(0%)
		Kourou, Montagne des Singes, 2016 (#5)	5°04'2"N 52°41'5"W	Old-growth forests	Vegetation	Larvae	5	0	(0%)
15	*A. scalpturatum* Neumann, 1906	Petit-Saut, montagne Plomb, 2016 (#35)	5°04'2"N 52°58'5"W	Old-growth forests	Human	Adult	1	0	(0%)
		Kourou, CSG, 2016 (#11)	5°11'3"N 52°46'5"W	Old-growth forests	Human	Nymph	2	0	(0%)
		Kourou, Montagne des Singes, 2016 (#5)	5°04'2"N 52°41'5"W	Old-growth forests	Vegetation	Adult	1	0	(0%)
		Station des Nouragues, 2017 (#37)	4°05'4"N 52°40'6"W	Old-growth forests	Human	Larvae	3	0	(0%)
		Régina, Savane-Roche Virginie, 2016 (#10)	4°11'4"N 52°08'W	Old-growth forests	Vegetation	Adult	1	0	(0%)
16	*A. varium* Koch, 1844	Rémire-Montjloy, Sentier Loyola, 2017 (#48)	4°53'5"N 52°16'2"W	Degraded forest	Vegetation	Nymph	1	1	(100%)
		Matoury, Piste de la Mirande, 2016 (#1)	4°51'5"N 52°20'3"W	Degraded forest	Vegetation	Larvae	3	2	(67%)
		Matoury, Piste de la Mirande, 2017 (#43)	4°51'5"N 52°20'3"W	Degraded forest	Human	Nymph	1	0	(0%)
		Matoury, Larivot, 2016 (#16)	4°54'0"N 52°20'2"W	Degraded forest	Sloth (*Bradypus tridactylus*)	Adults	2	1	(50%)
		Rémire-Montjoly, Vidal, 2016 (#32)	4°52'4"N 52°17'5"W	Degraded forest	Sloth (*Bradypus tridactylus*)	Adult	1	1	(100%)
17	*Dermacentor nitens* Neumann, 1897	Macouria, N1 road PK 34 (#4)	5°00'4"N 52°32'4"W	Pasture	Horses (*Equus caballus*)	Larvae, nymphs and adults	97	0	(0%)
18 -	*Haemaphysalis juxtakochi* Cooley, 1946	Kourou, CSG, 2016 (#11)	5°11'3"N 52°46'5"W	Old-growth forests	Human	Nymph	1	0	(0%)
		Kourou, Montagne des Singes, 2016 (#5)	5°04'2"N 52°41'5"W	Old-growth forests	Vegetation	Nymphs	2	1	(50%)
		Petit-Saut, montagne Plomb, 2016 (#7)	5°04'2"N 52°58'5"W	Old-growth forests	Vegetation	Nymphs	5	2	(40%)
19	*Ixodes luciae* Senevet, 1940	Cayenne, Camp du tigre, 2017 (#46)	4°54'36"N 52°17'5"W	Degraded forest	Gray Four-eyed Opossum (*Philander opossum*) and Linnaeus's Mouse Opossum (*Marmosa murina*)	Larva and adult	2	2	(100%)
		Cayenne, Montagne Tigre, 2017 (#40)	4°54'36"N 52°17'6"W	Degraded forest	Gray Four-eyed Opossum (*Philander opossum*)	Adults	3	3	(100%)

		Rémire-Montjoly, Vidal, 2016 (#18)	4°52'4"N 52°17'5"W	Degraded forest	Large-headed rice rat (*Hylaeamys megacephalus*) and Gray Four-eyed Opossum (*Philander opossum*)	Nymph	1	1	(100%)
20	*Rhipicephalus microplus* (Canestrini, 1888)	Macouria, Matiti, 2016 (#3)	5°02'3"N 52°32'5"W	Pasture	Cattle (*Bos taurus indicus*) and vegetation	Larvae and adults	10	0	(0%)
21	*R. sanguineus* sensus lato (Latreille, 1806)	Kourou, rue Maurice Ravel, 2016 (#12)	5°09'6"N 52°38'5"W	Peri-urban	Dog (*Canis lupus familiaris*)	Adults	3	0	(0%)
		Rémire-Montjoly, Montravel, 2016 (#14)	4°54'4"N 52°15'4"W	Peri-urban	Dog (*Canis lupus familiaris*)	Adults	3	0	(0%)
Argasidae (soft ticks):									
22	*Ornithodoros capensis*sensus stricto Neumann, 1901	Grand Connétable island, 2016, (#9)	4°49'3"N 51°57'W	Seabird colony	Laughing gull nest (*Leucophaeus atricilla*)	Nymphs and adults	6	0	(0%)

7

Table S2. Genes and primers used for *Rickettsia* sequencing.

Genes	*Rickettsia* specie	Hypothetical product	Primers (5'-3')		Tm	Fragment size	References
16S rRNA	All	Small ribosomal subunit	R16SF1-	CGTGGGAATCTGCCCATCAG	55°C	Semi-nested PCR assay:	This study
			R16SR1-	CTCTGCGATCCGCRACCACC		1st round PCR: R16SF1/R16SR2: 1015bp	
			R16SF2-	CGCTGATGGATGAGCCCGCGTC		2nd round PCR: R16SF2/Cox16SR1: 869bp	
gltA	All	Citrate synthase	RickF1-	GTTCTCTTTCKGCATTTTATCC	56°C	Semi-nested PCR assay:	[1]
			RickF2-	GTTCTCTTTCKGCATTTTATCC		1st round PCR: RickF1/RickR1: 672bp	
			RickR1-	CATCTTTAAGAGCGATAGCTTCAAG		2nd round PCR: RickF2/RickR1: 645bp	
coxA	All	Cytochrome c oxidase subunit 1	RcoxAF2-	CCYGATATGGCATTTCCACGCC	55°C	Semi-nested PCR assay:	This study
			RcoxAR2-	AAGCACCGAGCGACATCGTA		1st round PCR: RcoxAF2/RcoxaAR2: 849bp	
			RcoxAR1-	ACATATGGTGAGCCCATACGAT		2nd round PCR: RcoxAF2/RcoxaAR1: 568bp	
atpA	Spotted fever group species	ATP synthase subunit alpha	RatpAF1-	ATCGGTGATAGRCAAACCGGA	55°C	Semi-nested PCR assay:	This study
			RatpAR2-	CGAACCTGCYACCTGCTTCATAG		1st round PCR: RatpAF1/RatpAR2: 624bp	
			RatpAF2-	ACGGCGATAGCSGTTGATACT		2nd round PCR: RatpAF2/RatpAR2: 600bp	
	Bellii group species		RatpAbelliiF1-	GCTCCTGGTATAATCGATAG	55°C	Semi-nested PCR assay:	This study
			RatpAR2-	CGAACCTGCYACCTGCTTCATAG		1st round PCR: RatpAbelliiF1/RatpAR2: 730bp	
			RatpAR1-	GTGCTGCAGAACCAACACGGCT		2nd round PCR: RatpAbelliiF1/RatpAR1: 699bp	
ompB	Spotted fever group species	Outer membrane protein B precursor	RompBF1-	GGCTGGACCTGAAGCTGGAGC	52°C	Nested PCR assay:	This study
			RompBR2-	GCATCAGGTCTTATGCTTGCAC		1st round PCR: RompBF1/RompBR2: 792bp	
			RompBSFF2-	GCTTACGGTATATGGGCA		2nd round PCR: RompBSFF2/RompBR1: 644bp	
			RompBR1-	GTCCATCTAACTGAGACTGAG			
	Bellii group species		RompBbelliiF1-	CAGTGGTGCAGCAGAAG	53°C	Semi-nested PCR assay:	This study
			RompBbelliiR2-	GGCGGACTTTAACAGTACCCTG		1st round PCR: RompBbelliiF1/RompBbelliiR2: 1373bp	
			RompBbelliiF2-	GGCATCAAGTGAAGGCGGTG		2nd round PCR: RompBbelliiF2/RompBbelliiR2:: 859bp	

Reference

1. Duron, O. *et al*. Evolutionary changes in symbiont community structure in ticks. *Mol Ecol* **26**, 2905–2921 (2017). doi:10.1111/mec.14094

Figure legends

Figure S1. Phylogeny of *Rickettsia* constructed using maximum-likelihood (ML) estimations based on 16S rDNA sequences (730 unambiguously aligned nucleotide sites; best-fit approximation for the evolutionary model: TN93+G). Sequences from *Rickettsia* characterized in this study are shown in red. Only one 16S rDNA sequence per *Rickettsia* genotype and per tick species is shown. Sequences from representative *Rickettsia* groups, species and strains available in GenBank were also added to the analysis. Bacterial name, host species and GenBank accession numbers are shown on the tree. Branch numbers indicate percentage bootstrap support for major branches (1000 replicates; only bootstrap values >70% are shown). The scale bar is in units of substitution/site.

Figure S2. Phylogeny of *Rickettsia* constructed using maximum-likelihood (ML) estimations based on *atpA* gene sequences (567 unambiguously aligned nucleotide sites; best-fit approximation for the evolutionary model: GTR+G). Sequences from *Rickettsia* characterized in this study are shown in red. Only one *atpA* sequence per *Rickettsia* genotype and per tick species is shown. Sequences from representative *Rickettsia* groups, species and strains available in GenBank were also added to the analysis. Bacterial name, host species and GenBank accession numbers are shown on the tree. Branch numbers indicate percentage bootstrap support for major branches (1000 replicates; only bootstrap values >70% are shown). The scale bar is in units of substitution/site.

Figure S3. Phylogeny of *Rickettsia* constructed using maximum-likelihood (ML) estimations based on *ompB* gene sequences (644 unambiguously aligned nucleotide sites; best-fit approximation for the evolutionary model: GTR+G). Sequences from *Rickettsia* characterized in this study are shown in red. Only one *ompB* sequence per *Rickettsia* genotype and per tick species is shown. Sequences from representative *Rickettsia* groups, species and

strains available in GenBank were also added to the analysis. Bacterial name, host species and GenBank accession numbers are shown on the tree. Branch numbers indicate percentage bootstrap support for major branches (1000 replicates; only bootstrap values >70% are shown). The scale bar is in units of substitution/site.

Figure S4. Phylogeny of *Rickettsia* constructed using maximum-likelihood (ML) estimations based on *coxA* gene sequences (546 unambiguously aligned nucleotide sites; best-fit approximation for the evolutionary model: GTR+G). Sequences from *Rickettsia* characterized in this study are shown in red. Only one *coxA* sequence per *Rickettsia* genotype and per tick species is shown. Sequences from representative *Rickettsia* groups, species and strains available in GenBank were also added to the analysis. Bacterial name, host species and GenBank accession numbers are shown on the tree. Branch numbers indicate percentage bootstrap support for major branches (1000 replicates; only bootstrap values >70% are shown). The scale bar is in units of substitution/site.

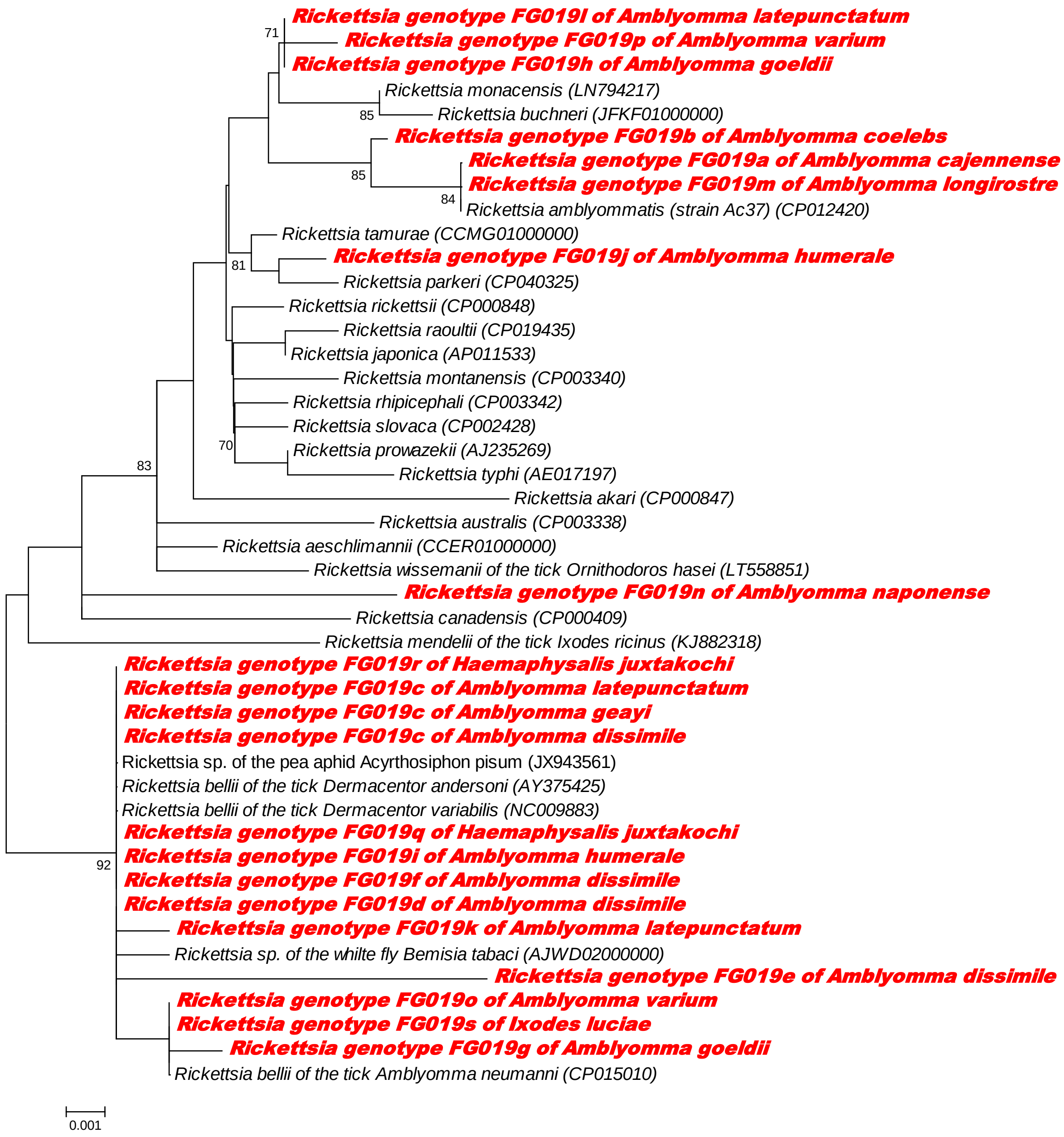

Rickettsia genotype FG019l of Amblyomma latepunctatum
Rickettsia genotype FG019p of Amblyomma varium
Rickettsia genotype FG019h of Amblyomma goeldii
71
Rickettsia monacensis (LN794217)
85
Rickettsia buchneri (JFKF01000000)
Rickettsia genotype FG019b of Amblyomma coelebs
Rickettsia genotype FG019a of Amblyomma cajennense
85
Rickettsia genotype FG019m of Amblyomma longirostre
84
Rickettsia amblyommatis (strain Ac37) (CP012420)
Rickettsia tamurae (CCMG01000000)
Rickettsia genotype FG019j of Amblyomma humerale
81
Rickettsia parkeri (CP040325)
Rickettsia rickettsii (CP000848)
Rickettsia raoultii (CP019435)
Rickettsia japonica (AP011533)
Rickettsia montanensis (CP003340)
Rickettsia rhipicephali (CP003342)
Rickettsia slovaca (CP002428)
70
Rickettsia prowazekii (AJ235269)
Rickettsia typhi (AE017197)
83
Rickettsia akari (CP000847)
Rickettsia australis (CP003338)
Rickettsia aeschlimannii (CCER01000000)
Rickettsia wissemanii of the tick Ornithodoros hasei (LT558851)
Rickettsia genotype FG019n of Amblyomma naponense
Rickettsia canadensis (CP000409)
Rickettsia mendelii of the tick Ixodes ricinus (KJ882318)
Rickettsia genotype FG019r of Haemaphysalis juxtakochi
Rickettsia genotype FG019c of Amblyomma latepunctatum
Rickettsia genotype FG019c of Amblyomma geayi
Rickettsia genotype FG019c of Amblyomma dissimile
Rickettsia sp. of the pea aphid Acyrthosiphon pisum (JX943561)
Rickettsia bellii of the tick Dermacentor andersoni (AY375425)
Rickettsia bellii of the tick Dermacentor variabilis (NC009883)
Rickettsia genotype FG019q of Haemaphysalis juxtakochi
92
Rickettsia genotype FG019i of Amblyomma humerale
Rickettsia genotype FG019f of Amblyomma dissimile
Rickettsia genotype FG019d of Amblyomma dissimile
Rickettsia genotype FG019k of Amblyomma latepunctatum
Rickettsia sp. of the whilte fly Bemisia tabaci (AJWD02000000)
Rickettsia genotype FG019e of Amblyomma dissimile
Rickettsia genotype FG019o of Amblyomma varium
Rickettsia genotype FG019s of Ixodes luciae
Rickettsia genotype FG019g of Amblyomma goeldii
Rickettsia bellii of the tick Amblyomma neumanni (CP015010)
0.001

Figure S1

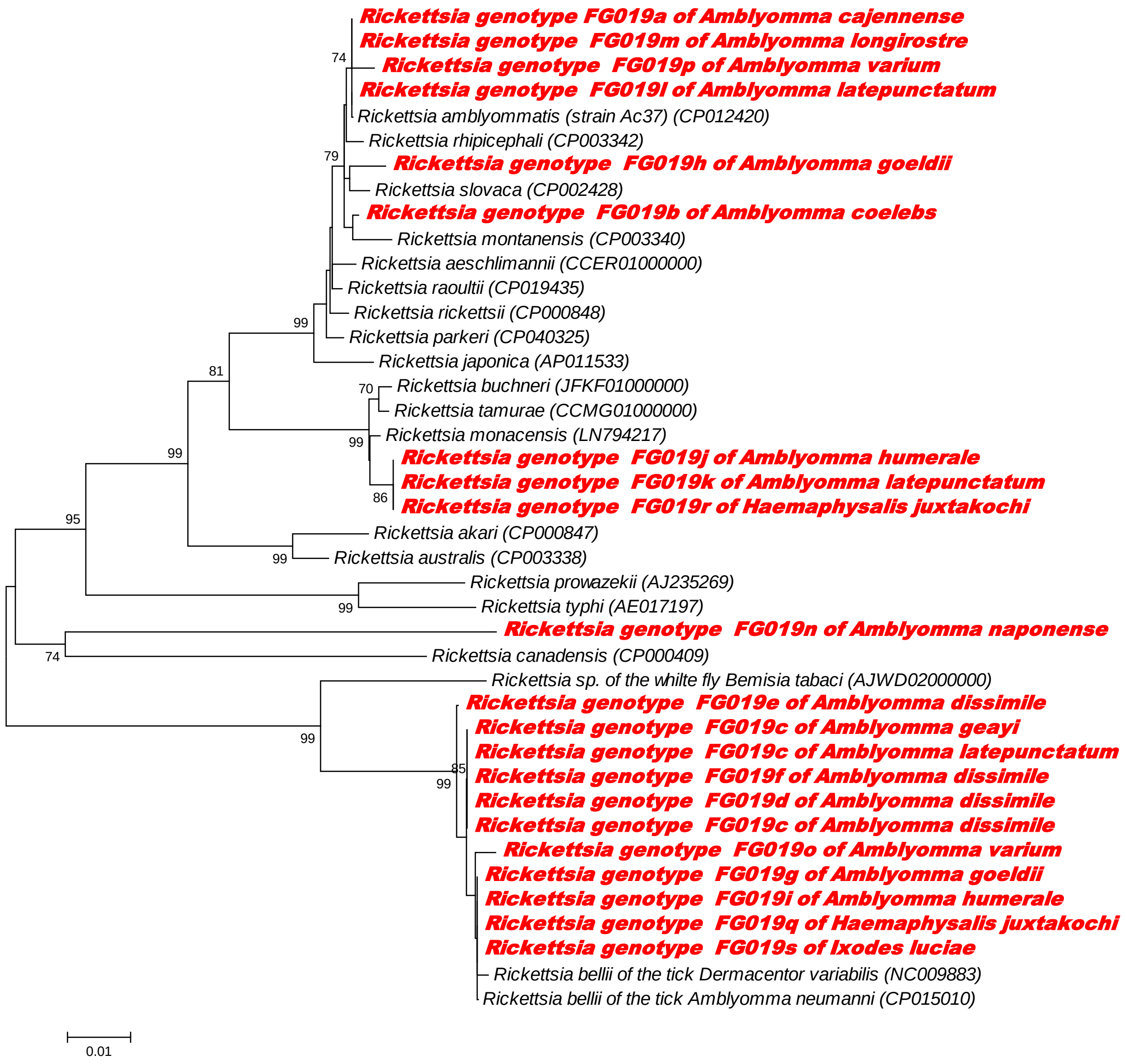
Rickettsia genotype FG019a of Amblyomma cajennense
Rickettsia genotype FG019m of Amblyomma longirostre
Rickettsia genotype FG019p of Amblyomma varium
Rickettsia genotype FG019l of Amblyomma latepunctatum
Rickettsia amblyommatis (strain Ac37) (CP012420)
Rickettsia rhipicephali (CP003342)
Rickettsia genotype FG019h of Amblyomma goeldii
Rickettsia slovaca (CP002428)
Rickettsia genotype FG019b of Amblyomma coelebs
Rickettsia montanensis (CP003340)
Rickettsia aeschlimannii (CCER01000000)
Rickettsia raoultii (CP019435)
Rickettsia rickettsii (CP000848)
Rickettsia parkeri (CP040325)
Rickettsia japonica (AP011533)
Rickettsia buchneri (JFKF01000000)
Rickettsia tamurae (CCMG01000000)
Rickettsia monacensis (LN794217)
Rickettsia genotype FG019j of Amblyomma humerale
Rickettsia genotype FG019k of Amblyomma latepunctatum
Rickettsia genotype FG019r of Haemaphysalis juxtakochi
Rickettsia akari (CP000847)
Rickettsia australis (CP003338)
Rickettsia prowazekii (AJ235269)
Rickettsia typhi (AE017197)
Rickettsia genotype FG019n of Amblyomma naponense
Rickettsia canadensis (CP000409)
Rickettsia sp. of the whilte fly Bemisia tabaci (AJWD02000000)
Rickettsia genotype FG019e of Amblyomma dissimile
Rickettsia genotype FG019c of Amblyomma geayi
Rickettsia genotype FG019c of Amblyomma latepunctatum
Rickettsia genotype FG019f of Amblyomma dissimile
Rickettsia genotype FG019d of Amblyomma dissimile
Rickettsia genotype FG019c of Amblyomma dissimile
Rickettsia genotype FG019o of Amblyomma varium
Rickettsia genotype FG019g of Amblyomma goeldii
Rickettsia genotype FG019i of Amblyomma humerale
Rickettsia genotype FG019q of Haemaphysalis juxtakochi
Rickettsia genotype FG019s of Ixodes luciae
Rickettsia bellii of the tick Dermacentor variabilis (NC009883)
Rickettsia bellii of the tick Amblyomma neumanni (CP015010)
74
79
99
81
70
99
86
99
95
99
99
74
99
85
99
0.01

Figure S2

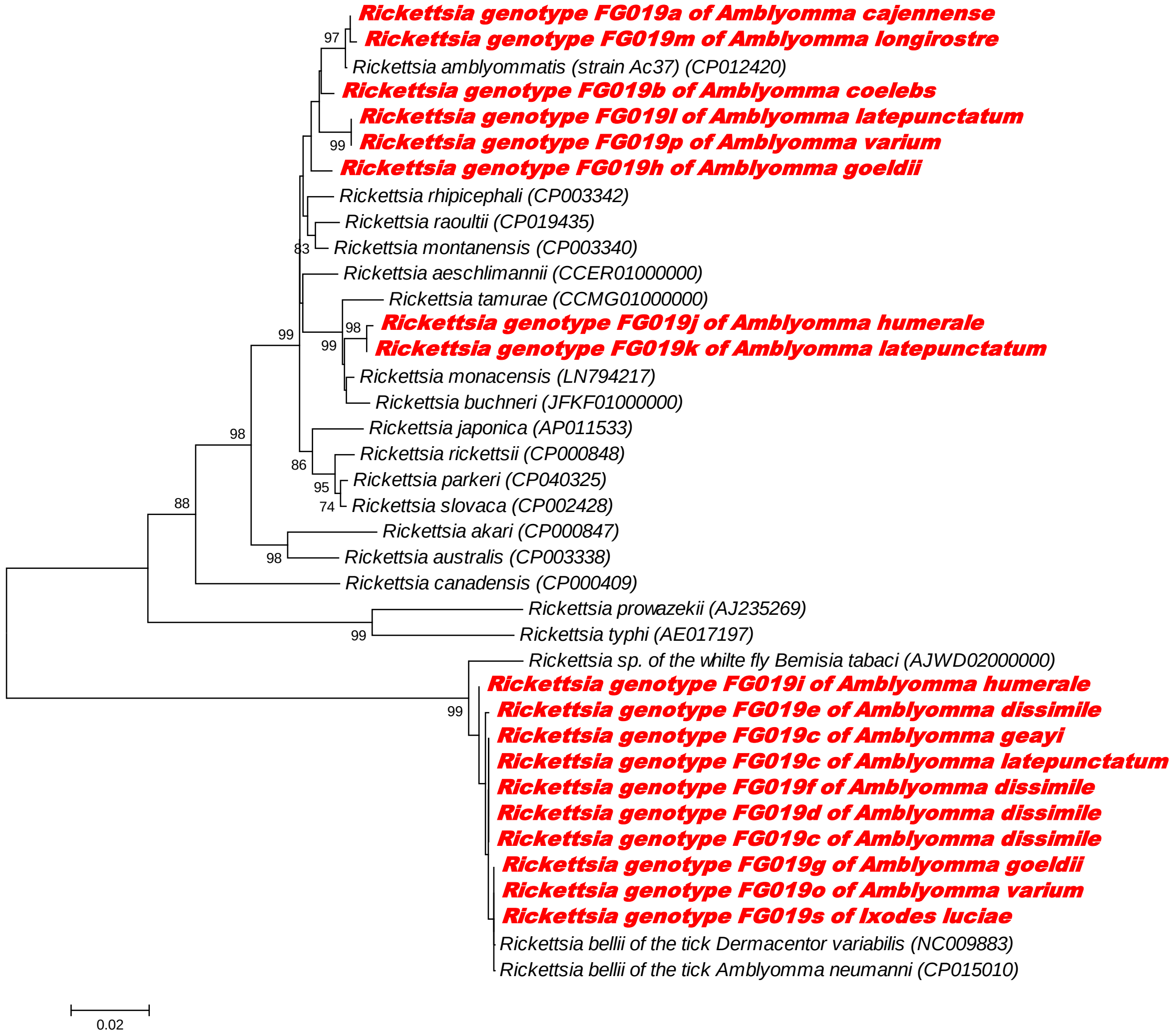
Rickettsia genotype FG019a of Amblyomma cajennense
97
Rickettsia genotype FG019m of Amblyomma longirostre
Rickettsia amblyommatis (strain Ac37) (CP012420)
Rickettsia genotype FG019b of Amblyomma coelebs
Rickettsia genotype FG019l of Amblyomma latepunctatum
99
Rickettsia genotype FG019p of Amblyomma varium
Rickettsia genotype FG019h of Amblyomma goeldii
Rickettsia rhipicephali (CP003342)
Rickettsia raoultii (CP019435)
83
Rickettsia montanensis (CP003340)
Rickettsia aeschlimannii (CCER01000000)
Rickettsia tamurae (CCMG01000000)
98
Rickettsia genotype FG019j of Amblyomma humerale
99
Rickettsia genotype FG019k of Amblyomma latepunctatum
99
Rickettsia monacensis (LN794217)
Rickettsia buchneri (JFKF01000000)
98
Rickettsia japonica (AP011533)
86
Rickettsia rickettsii (CP000848)
95
Rickettsia parkeri (CP040325)
74
Rickettsia slovaca (CP002428)
88
Rickettsia akari (CP000847)
98
Rickettsia australis (CP003338)
Rickettsia canadensis (CP000409)
Rickettsia prowazekii (AJ235269)
99
Rickettsia typhi (AE017197)
Rickettsia sp. of the whilte fly Bemisia tabaci (AJWD02000000)
Rickettsia genotype FG019i of Amblyomma humerale
99
Rickettsia genotype FG019e of Amblyomma dissimile
Rickettsia genotype FG019c of Amblyomma geayi
Rickettsia genotype FG019c of Amblyomma latepunctatum
Rickettsia genotype FG019f of Amblyomma dissimile
Rickettsia genotype FG019d of Amblyomma dissimile
Rickettsia genotype FG019c of Amblyomma dissimile
Rickettsia genotype FG019g of Amblyomma goeldii
Rickettsia genotype FG019o of Amblyomma varium
Rickettsia genotype FG019s of Ixodes luciae
Rickettsia bellii of the tick Dermacentor variabilis (NC009883)
Rickettsia bellii of the tick Amblyomma neumanni (CP015010)
0.02

Figure S3

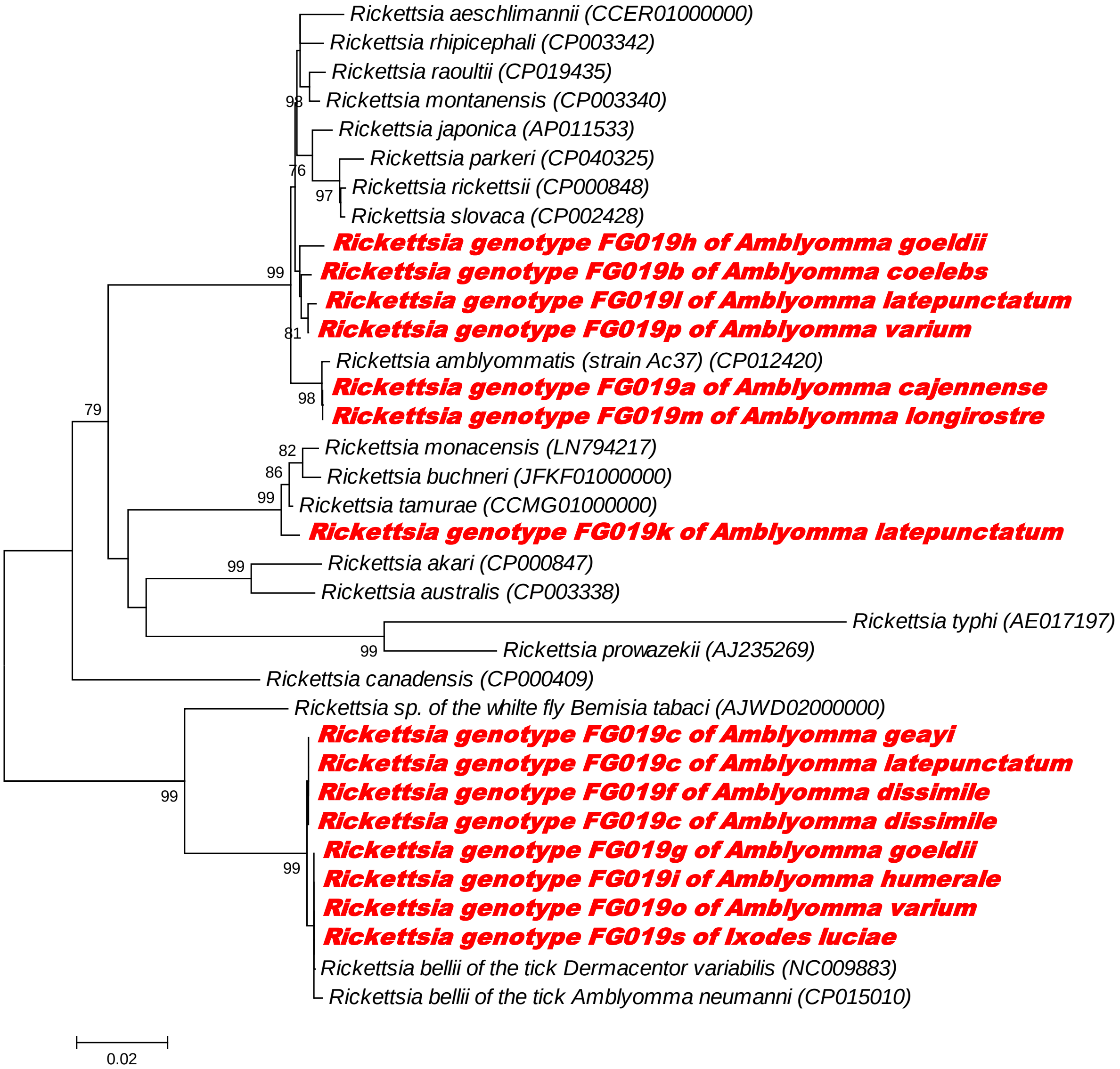
Rickettsia aeschlimannii (CCER01000000)
Rickettsia rhipicephali (CP003342)
Rickettsia raoultii (CP019435)
Rickettsia montanensis (CP003340)
Rickettsia japonica (AP011533)
Rickettsia parkeri (CP040325)
Rickettsia rickettsii (CP000848)
Rickettsia slovaca (CP002428)
Rickettsia genotype FG019h of Amblyomma goeldii
Rickettsia genotype FG019b of Amblyomma coelebs
Rickettsia genotype FG019l of Amblyomma latepunctatum
Rickettsia genotype FG019p of Amblyomma varium
Rickettsia amblyommatis (strain Ac37) (CP012420)
Rickettsia genotype FG019a of Amblyomma cajennense
Rickettsia genotype FG019m of Amblyomma longirostre
Rickettsia monacensis (LN794217)
Rickettsia buchneri (JFKF01000000)
Rickettsia tamurae (CCMG01000000)
Rickettsia genotype FG019k of Amblyomma latepunctatum
Rickettsia akari (CP000847)
Rickettsia australis (CP003338)
Rickettsia typhi (AE017197)
Rickettsia prowazekii (AJ235269)
Rickettsia canadensis (CP000409)
Rickettsia sp. of the whilte fly Bemisia tabaci (AJWD02000000)
Rickettsia genotype FG019c of Amblyomma geayi
Rickettsia genotype FG019c of Amblyomma latepunctatum
Rickettsia genotype FG019f of Amblyomma dissimile
Rickettsia genotype FG019c of Amblyomma dissimile
Rickettsia genotype FG019g of Amblyomma goeldii
Rickettsia genotype FG019i of Amblyomma humerale
Rickettsia genotype FG019o of Amblyomma varium
Rickettsia genotype FG019s of Ixodes luciae
Rickettsia bellii of the tick Dermacentor variabilis (NC009883)
Rickettsia bellii of the tick Amblyomma neumanni (CP015010)
98
76
97
99
81
98
79
82
86
99
99
99
99
99
0.02

Figure S4
